# Characterization of Key Aroma Compounds in Fermented Bamboo Shoots Using Gas Chromatography-Olfactometry-Mass Spectrometry, Odor Activity Values, and Aroma Recombination Experiments

**DOI:** 10.3390/foods11142106

**Published:** 2022-07-15

**Authors:** Shubo Li, Yufeng Tian, Minghao Sun, Jiaojiao Liu, Yunxia Bai, Xiaoling Liu, Yuan Guo

**Affiliations:** 1 College of Light Industry and Food Engineering, Guangxi University, Nanning 530004, China; shubo1207@gxu.edu.cn (S.L.); 1916392008@st.gxu.edu.cn (Y.T.); sunminghao1997@163.com (M.S.); 2116392006@st.gxu.edu.cn (J.L.); 2116392018@st.gxu.edu.cn (Y.B.); tianyufeng010@163.com (X.L.); 2 National Engineering Research Center for Non-Food Biorefinery, Guangxi Academy of Sciences, Nanning 530004, China

**Keywords:** Guangxi fermented bamboo shoots, key aroma compounds, HS-SPME-GC-O-MS, OAVs, recombination experiment

## Abstract

Guangxi fermented bamboo shoots (GFBS) are widely appreciated by consumers in China because of their unique aroma. In this study, the dominant aroma compounds of GFBS were investigated using gas chromatography-olfactometry-mass spectrometry, odor-activity values, and aroma recombination. The results show that 70 aroma compounds, including alcohols, esters, aldehydes, acids, phenols, ethers, ketones, alkenes, benzene derivatives, and furans, were identified in GFBS. Among them, 15 aroma compounds with odor-activity values (OAVs) > 1 were identified. Aroma-recombination-omission experiments and sensory evaluation demonstrated that octanal, (E)-2-octenal, acetic acid, guaiacol, phenylethyl alcohol, creosol, 4-ethylguaiacol, and p-cresol significantly contributed to the characteristic aroma of GFBS. Most importantly, p-cresol (34,997.95 ≤ OAV ≤ 71,409.51) and acetic acid (2155.79 ≤ OAV ≤ 3872.09) significantly contributed to its aroma (*p* < 0.001). The major aroma profile of GFBS included a strong fermented odor, which was pungent and sour. This study provides a theoretical basis for improving the flavor of GFBS.

## 1. Introduction

Fermented bamboo shoots (FBS) have tremendous health benefits, including such properties as anti-cancer, anti-oxidant, anti-aging, cardioprotective, weight loss, and probiotic, to name a few. In addition, FBS are an important functional food with both industrial and economic value [1,2,3,4]. Nowadays, their value is appreciated worldwide. In India, fermented bamboo shoots are referred to as “green gold” for their high nutritional value [3], and they have become part of an everyday diet in Taiwan, China [5]. GFBS is a unique FBS produced in the Guangxi Zhuang Autonomous Region, China. GFBS is also the main ingredient and flavor of many characteristically Chinese foods in Guangxi (in a similar manner as Luosifen, which is a kind of famous noodles). Notably, aroma is regarded as one of the most important factors for evaluating the quality and consumer acceptance of GFBS.

Despite this, only a few studies have been conducted on the structure of the characteristic flavor components of GFBS. For example, Guo et al. [6] compared the extraction effects of liquid–liquid extraction, simultaneous distillation extraction, and headspace solid-phase microextraction (HS-SPME) using gas chromatography-mass spectrometry (GC-MS). The results showed that HS-SPME was more suitable for extracting odorants from fermented bamboo shoots resulting in the largest peak area and the greatest number of substances extracted. In addition, 41 volatile compounds were detected, including phenol, acid, alcohol, and others (68.13, 15.03, 14.22, and 2.62%, respectively), among which p-cresol, phenol, acetic acid, and ethanol were the main volatiles. Zheng et al. [7] detected 86 types of compounds in GFBS using the HS-SPME method and revealed 18 types of amines, 13 alcohols, 7 esters, and 2 aldehydes as the main volatile components, according to the relative percentage peak area. Guo et al. [8] identified and quantified 43 volatiles in GFBS using HS-SPME-GC-MS and detected 16 compounds, including 5 alcohols, 4 aromatics, 4 aldehydes, 2 carboxylic acids, and 1 ester, all of which had high odor activity values (OAV) > 1, according to the internal standard method. Among these, 1-octen-3-ol, geraniol, p-cresol, nonanal, decanal, (E)-2-nonenal, phenylethyl alcohol, and octanoic acid were found to have high flavor dilution (≥243), which may explain the potent odorants that form the unique flavor of GFBS. However, the aroma components of GFBS are known because of semi-quantitative analysis using the internal standard method, which cannot accurately determine the key aroma components in the food matrix [8,9]. It has not yet been confirmed which aroma compounds constitute the characteristic aroma of GFBS. Therefore, further research is required to confirm the key aroma-active compounds in GFBS for quality control and flavor enhancement.

At present, a comprehensive approach, including the identification of key aroma compounds using GC-O and OAV analysis [10] and aroma recombination and omission experiments, have been used to more accurately characterize and confirm the key odor compounds in food matrices [11,12]. Liu et al. reported that 18 important odorants with OAVs > 1 were screened out from Beijing roast duck using semi-quantification, according to internal standards, leaving 9 proven key aroma compounds [13]. Similarly, 24 important aroma compounds were identified in Zhuhoujiang using odor intensity and OAV analysis, and aroma-recombination-omission experiments demonstrated that four aroma compounds significantly contributed to the characteristic aroma of Zhuhoujiang [14]. Therefore, the aim of this study was to clarify the aroma characteristics of GFBS using sensory evaluation, to validate the important aroma compounds using GC-O and OAV analysis, and to confirm the key aroma compounds using aroma recombination and omission experiments. This study provides a better understanding of the key aroma compounds in GFBS and provides a theoretical basis for improving the flavor of GFBS.

## 2. Materials and Methods

### 2.1. Chemicals and Materials

Several chemical standards were purchased from Sigma-Aldrich (Shanghai, China): propanal, ethyl acetate, octanal, nonanal, 1-octen-3-ol, (E)-2-octenal, acetic acid, benzaldehyde, 1-nonanol, methyl salicylate, guaiacol, geranylacetone, phenylethyl alcohol, creosol, 4-ethylguaiacol, anisaldehyde, p-cresol, and 4-ethyl-phenol. The internal standard (2-methyl-3-heptanone) and a C7-C40 n-alkane mixture were purchased from Sigma-Aldrich. Chemical standards and internal standards were of a high-purity grade (GC grade ≥ 97% purity). GFBS were collected from several fermentation workshops (in the City of Liuzhou (LZ), Nanning (NN), Guilin (GL), and Baise (BS), Guangxi Zhuang Autonomous Region, China), in which the fresh shoots of *Dendrocalamus latiflorus* Munro were peeled and then steeped in mountain spring water for anaerobically fermenting in a jar for 30 d at 25 °C. Their basic information is listed in Appendix A Table A1. All samples were wrapped in nylon/polyethylene and stored at −20 °C until analysis.

### 2.2. Extraction of Volatile Compounds Using HS-SPME

HS-SPME was carried out according to the methods described by Guo et al. [8] with minor modifications. Each GFBS sample was mixed with distilled water at a ratio of 1:1 (*w*/*w*) and homogenized. The homogenized sample (4.0 g) and NaCl (0.8 g) were placed in a 20 mL headspace vial, and 1.5 µL of 2-methyl-3-heptanone (internal standard, 1.65 µg/µL) in absolute methanol was added. First, the vial was sealed with a polytetrafluoroethylene silicon stopper and preheated at 50 °C for 30 min. Then, the SPME coating fiber (50/30 µm CAR/PDMS/DVB, Supelco, Inc., Bellefonte, PA, USA) was exposed to the HS 1 cm above the solution surface to extract the volatile compounds at 50 °C for 30 min. Finally, the coating fiber was quickly inserted into the GC injection port (250 °C) for 3 min for desorption and separation. After each analysis, the fiber was inserted into a heater at 250 °C for 20 min to ensure that there was no residue.

### 2.3. Identification of Aroma Compounds Using GC-O-MS

GC-MS analysis was performed using a GC-MS (7890 B GC System, 5977A MSD); GC-O analysis was performed using a GC-MS (7890 B GC System, 5977A MSD) equipped with an olfactory detector port (ODP) (Gerstal, Mulheim an der Ruhr, Germany).

A 122-5532UIHP-INNOWAX capillary column (60 m × 250 µm × 0.25 µm, Agilent Technologies, Santa Clara, CA, USA) was used, and the column temperature was maintained at 40 °C for 2 min, ramped to 140 °C at a rate of 6 °C/min, held for 5 min, ramped to 150 °C at a rate of 3 °C/min with a 1 min holding time, then ramped to 197 °C at a rate of 5 °C/min, held for 2 min, ramped to 205 °C at a rate of 1 °C/min, and finally ramped to 240 °C at a rate of 7 °C/min and held for 15 min. The GC effluent was split 1:1 between the MSD and ODP through a T-type splitter. Electron-impact mass spectra were generated at 70 eV, with an m/z scan range of 40–400 amu. The ion source, detector interface, and olfactometer temperatures were 230, 250, and 200 °C, respectively. Aroma compounds were identified by comparison with a mass-spectrometry library (NIST 14.0 mass-spectrometry database), retention indices (RIs), odor qualities, and authentic flavor standards. The retention indices (RIs) of unknown aroma compounds were calculated from the retention times of n-alkanes (C7–C40) according to the improved Kovats method. Then, the aroma compounds were determined by comparing RIs to n-alkanes (C7−C40) reported in the literature. The odor qualities of the aroma compounds were determined by experienced panelists who were familiar with odor descriptions and solutions of artificial odorant. Authentic flavor standards were used as external standards using GC-O-MS. Each GFBS sample was tested in triplicate.

### 2.4. Quantitative Assessment of Aroma Compounds

The aroma compounds in GFBS were semi-quantified using 2-methyl-3-heptanone as an internal standard. Briefly, 1.5 µL of 2-methyl-3-heptanone was added to the sample and analyzed using GC-MS. The concentration of aroma compounds was calculated according to the ratio between the peak area and the concentration of 2-methyl-3-heptanone.

The aroma compounds identified through GC-MS-O were quantified, according to the literature, with several modifications [13,15,16]. Prior to quantification, an odorless artificial matrix was prepared by further treatment with GFBS. The sample was eluted with an organic solvent using HS-SPME-GC-O-MS until nothing was detected. The specific method was as follows: first, 1-butanol and di-isopropyl ether (B-DIPE) were added to the sample (1-butanol, B-DIPE, and sampled at a ratio of 2:3:2.5, *w*/*w*/*w*). Then, the mixture was shaken for 8 h, and the organic solvent was removed. This was repeated three times. Furthermore, the organic solvent in the mixture was removed by rotary evaporation at least three times. Samples were subsequently treated with liquid nitrogen (99.99%) and frozen in an FD-1D-5D vacuum freeze dryer (Beijing Biocool Lab Apparatus Co., Ltd., Beijing, China) at −60 °C for 48 h.

Based on semi-quantification, aroma compounds with OAVs > 1 were quantified using calibration curves [14]. The calibration curve was formed as follows: various concentrations (acetic acid: 12.5–1250 g/L; p-cresol and ethyl acetate: 0.09–20 g/L; propanal and octanal: 5 mg/L–5 g/L; methyl salicylate, geranylacetone, and 1-octen-3-ol: 0.2 mg/L–0.5 g/L; and others: 1 mg/L–1 g/L) of authentic flavor standards containing internal standard were added to an artificial odorless matrix using GC-selected-ion monitoring.

### 2.5. OAVs

The OAVs of the quantified compounds on the calibration curves were calculated using the following formula: $OAV=C_{i}/OT_{i}$, where $C_{i}$ is the concentration of compound I, and $OT_{i}$ is the odor threshold value in the water of compound *i*.

### 2.6. Quantitative Descriptive Sensory Analysis

Quantitative descriptive sensory analysis was carried out in a sensory room (the room temperature was 25 ± 1 °C), which was followed by ISO 8589 standard [17]. Nine sensory evaluators (five female and four male) who were 20–30 years old were recruited to perform GFBS sensory evaluation [18]. The sensory evaluators were selected from 53 candidates based on their performance in several olfactory tests and on the absence of olfactory allergies [19]. The sensory evaluators were trained with standard solutions and Guangxi fermented bamboo shoot products twice a week for six weeks to familiarize them with the tasks and underlying mechanisms of the testing and evaluation procedure. Each participant provided full informed consent. Five relevant flavor attributes—fermented, pungent, sour, alcoholic, and moldy—were selected after panel discussion. The intensity of each attribute was rated by the assessors on a 10-point scale (0: none to 10: very strong). The average results obtained from the sensory panel were ultimately mapped to a sensory profile.

### 2.7. Recombination and Omission Experiments

Aroma recombination was conducted to verify that the volatile compounds with high OAVs were the key odorants of GFBS. LZ-FBS have the richest flavor and were selected as the recombinant control. Recombination and omission experiments were performed as previously reported [20,21]. Recombination model 1 was formulated with an odorless matrix, authentic flavor standards with OAVs > 1, and ultrapure water. Recombination model 2 consisted of recombination model 1 with one flavor standard omitted. The aroma components of recombination model 2 were different from those of GFBS and recombination model 1, indicating that the missing aroma components of recombination model 2 are the key aroma components. Recombination model 3 consisted of key aroma compounds, an odorless matrix, and ultrapure water. Recombination model 3 was evaluated on the basis of whether it was consistent with GFBS according to panelists in a triangle test, according to the previously described method [21].

### 2.8. Statistical Analysis

All experimental results were based on three replicates. Statistical analysis was conducted using SPSS software (version 19.0; IBM Corporation, Armonk, NY, USA). The cluster heatmap of aroma compounds in GFBS was created using R software (version 4.0.2; R Foundation for statistical computing, Vienna, Austria) with the heatmap package. Results are expressed as mean ± standard deviation from three measurements.

## 3. Results

### 3.1. Sensory Analysis

**Figure 1:**
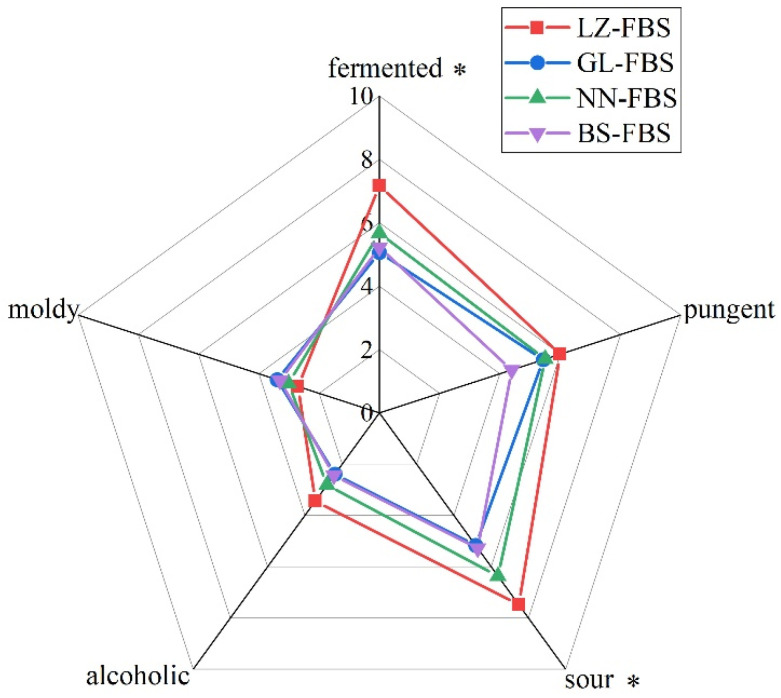
Sensory characteristics of Guangxi fermented bamboo shoots (GFBS) from different regions of Guangxi. * significant (*p* ≤ 0.05).

Quantitative description analysis was performed to determine the aroma characteristics of GFBS from the four regions of Guangxi. As shown in Figure 1, the aroma profiles of GFBS were described using five attributes, namely “fermented”, “pungent”, “sour”, “alcoholic”, and “moldy”. Analysis of variance was employed to distinguish differences in sensory evaluation scores, and the results showed that the intensity of two (fermented and sour) attributes significantly differed among the different GFBS samples (*p* < 0.05). The BS-FBS samples only scored highly in the “moldy” category and had very low scores for the other four sensory attributes. This might be due to the relatively high phenol content and low levels of other aroma-active compounds. NN-FBS and GL-FBS had significantly different “sour” scores but similar scores for the other attributes. In addition, LZ-FBS was characterized most strongly by a sour note with a score of 7.48, followed by “fermented” (7.17), “pungent” (6.00), and “alcoholic” (3.44).

### 3.2. Identification and Quantification of GFBS with GC-MS

As shown in Table 1, 70 aroma compounds were identified from GFBS from four regions of the Guangxi Zhuang Autonomous Region. These compounds were divided into ten groups: alcohols, esters, aldehydes, acids, phenols, ethers, ketones, alkenes, benzene derivatives, and furans. Among these, the highest number of chemical components detected in one group was alcohols; 13 to 16 alcohols were detected in LZ-FBS, GL-FBS, NN-FBS, and BS-GFBS. More importantly, the chemical class with the highest content was phenols, accounting for 59.8–76.16% in LZ-FBS, GL-FBS, NN-FBS, and BS-FBS. Compared to other GFBS samples, 57 aroma compounds were detected in the LZ-FBS, including 16 alcohols, 12 esters, 8 aldehydes, 5 acids, 8 phenols, 2 ethers, 3 ketones, and 3 others.

**Figure 2:**
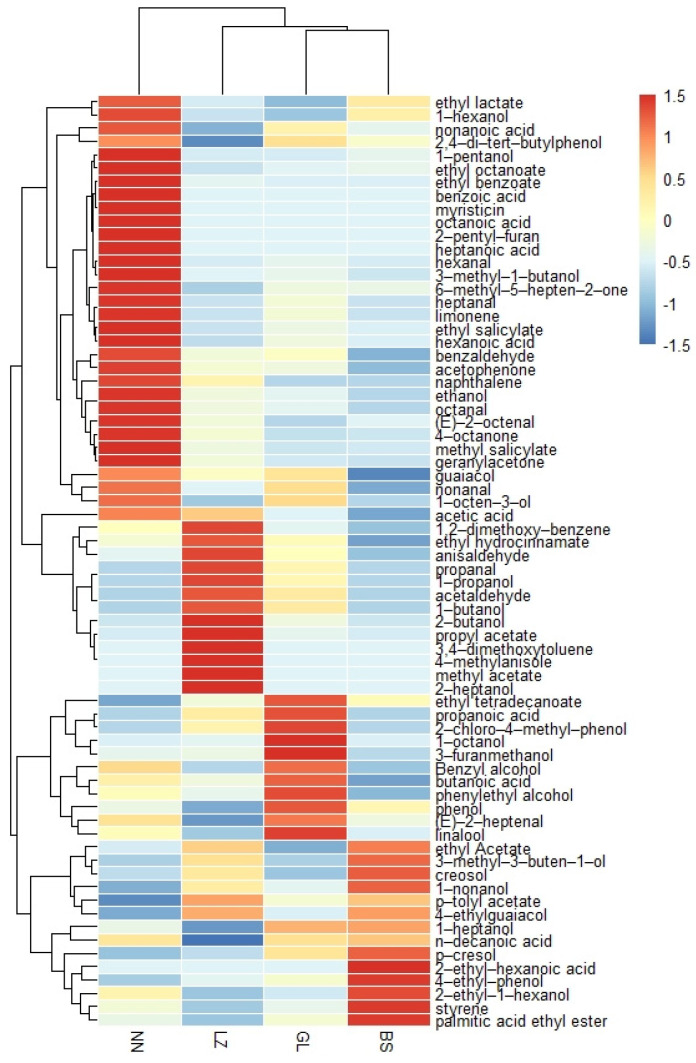
Heatmap analysis of aroma compounds in GFBS.

The contents of the aroma compounds in GFBS are summarized in Table 1. Propanal (9.39–22.06 ng/g), ethyl acetate (57.35–251.16 ng/g), hexanal (8.4–133.41 ng/g), octanal (1.96–12.13 ng/g), nonanal (5.93–16.44 ng/g), 1-octen-3-ol (8.04–93.01 ng/g), (E)-2-octenal (2–17.5 ng/g), acetic acid (2005.91–3710.7 ng/g), benzaldehyde (28.87–184.72 ng/g), 1-nonanol (9.97–11.85 ng/g), methyl salicylate (15.77–174.04 ng/g), guaiacol (51.22–143.74 ng/g), phenylethyl alcohol (22.77–277.87 ng/g), creosol (307.7–913.7 ng/g), 4-ethylguaiacol (69.04–235.61 ng/g), anisaldehyde (3.49–6.8 ng/g), p-cresol (15435.89–21323.09 ng/g), and 4-ethylphenol (64.89–167.54 ng/g) were detected. These compounds have low threshold values and were therefore considered the major aroma compounds. Meanwhile, ethanol (1930.17–2719.03 ng/g), ethyl lactate (60.05–423.33 ng/g), phenol (305.51–5757.22 ng/g), and nonanoic acid (182.91–530.72 ng/g) were found in high concentrations in GFBS from four areas of Guangxi. To distinguish GFBS from different regions of Guangxi intuitively, according to their aroma components, a heatmap (Figure 2) was generated using cluster analysis.

### 3.3. Identification of Aroma-Active Compounds by GC-MS-OSME and Their OAV Analysis

OSME is widely used to determine the compounds responsible for aroma perception. The results of the sensory evaluation demonstrated that LZ-FBS had the richest aroma and was most representative of GFBS in general. The LZ-FBS was therefore used for GC-O analysis, which revealed 18 aroma compounds. As shown in Table 1, the perceptible aroma compounds were propanal, ethyl acetate, octanal, nonanal, 1-octen-3-ol, (E)-2-octenal, acetic acid, benzaldehyde, 1-nonanol, methyl salicylate, guaiacol, geranylacetone, phenylethyl alcohol, creosol, 4-ethylguaiacol, anisaldehyde, p-cresol, and 4-ethyl-phenol. These were considered the primary contributors to the aroma of GFBS.

The contribution of the individual aroma compounds to the overall aroma of GFBS depends not only on the content of the compounds but also on their OAVs. To accurately calculate the OAV values of each aroma compound, their concentrations were calculated according to the calibration curves. As shown in Table 2, the calibration curves and characteristic ion fragments (*m*/*z*) were determined. The correlation coefficients (R^2^ > 0.99) revealed high linearity for aroma compounds in GFBS.

As shown in Table 3, the OAVs of aromatic compounds in GFBS from the four regions all > 1 are as follows: octanal, 25.17–66.14; nonanal, 2.61–10.31; 1-octen-3-ol, 9.09–36.46; (E)-2-octenal, 3.87–10.12; acetic acid, 2155.79–3872.09; 1-nonanol, 2.91–3.13; guaiacol, 29.62–118.32; creosol, 14.44–41.8; 4-ethylguaiacol, 1.15–23.11; anisaldehyde, 3.7–4.05; p-cresol, 34997.95–71409.51; and 4-ethyl-phenol, 2.02–4.25. Propanal (15.83–32.83), ethyl acetate (9.75–40.03), and phenylethyl alcohol (1.64–5.33) also had high OAVs in most GFBS samples. It is important to note that the OAVs of acetic acid and p-cresol were much higher than those of the other compounds, and they were the most decisive compounds. In summary, 15 aroma compounds with OAVs > 1 were detected in GFBS.

### 3.4. Determination of Key Aroma Compounds Using Recombination Experiments

To evaluate the overall importance of each aroma compound, aroma omission was performed with 15 aroma compounds with OAVs > 1. A triangle test was used for sensory evaluation. After comparing the odor properties of recombinant samples and GFBS samples, the main characteristics were described using five sensory attributes: fermented, pungent, sour, alcoholic, and moldy.

**Figure 3:**
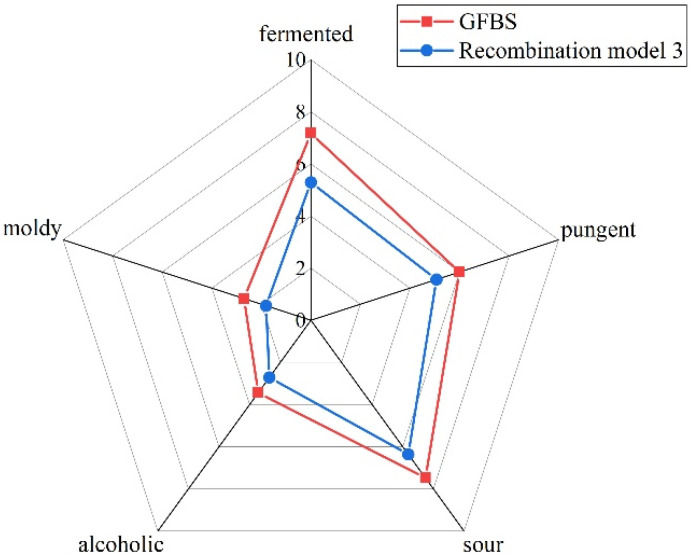
Aroma profiles of GFBS compared with aroma recombination model.

By comparing the aroma characteristics of recombinant model 1 and LZ-FBS, it was found that the sensory properties of the samples obtained by recombination of the 15 aroma compounds (recombinant model 1) were in accordance with the original sample. Fifteen omission models were prepared, and the contribution of each aroma compound was evaluated using a triangle test under the guidance of recombinant model 1. As shown in Table 4, eight aroma compounds, including aldehydes, acids, phenols, and alcohols, significantly influenced the aroma profile of the recombination model. Notably, acetic acid, guaiacol, and p-cresol exhibited highly significant differences (*p* ≤ 0.001) when they were omitted from the mixture. The omission of phenylethyl alcohol and creosol caused a highly significant difference (*p* ≤ 0.01). Moreover, when octanal, (E)-2-octenal, and 4-ethylguaiacol were omitted, the difference was also significant (*p* ≤ 0.05). We then carried out a recombination experiment with eight compounds that had a significant effect on the aroma of the profile of recombination model 1. The results are shown in Figure 3, and the aroma characteristics of recombinant model 3 were similar to those of GFBS. In terms of overall similarity, compared with the original GFBS, the recombined aroma model was rated 4.3 out of 5 points.

## 4. Discussion

In this study, 70 aroma compounds were identified from GFBS using GC-MS, and these were divided into 10 categories. Guo et al. identified 43 volatiles in GFBS using the HS-SPME method with a coating fiber (65 µm DVB/PDMS). In our study, we extracted volatiles from GFBS using a coating fiber (50/30 µm CAR/PDMS/DVB) and identified more volatile compounds.

### 4.1. Dominant Aroma Compounds in GFBS

As shown in Table 3 and Table 4, p-cresol had the highest OAVs and had a highly significant effect on the aroma of GFBS; this was therefore designated the most important aroma compound in GFBS. This is consistent with Guo et al. [6], in which p-cresol made the greatest contribution to odor composition in fermented bamboo shoots. In addition, p-cresol has been identified as an important aroma compound in fermented bacterial food [28]. Other phenolic compounds, including guaiacol, 4-ethylguaiacol, and creosol, were identified as the key aroma compounds in recombination tests. These phenolic compounds have also been reported to contribute to the characteristic aromas of some fermented foods. For example, guaiacol was reported to be the key aroma compound in Japanese miso [29], and 4-ethylguaiacol was identified as the key aroma compound of pixian broad bean paste (PBBP) and traditional Chinese soy sauce [9,27]. It has been reported that 4-ethylguaiacol and creosol contribute to spicy and smoky odors [30], which may play an important role in the production of pungent odors by GFBS. In summary, phenolic compounds were the dominant aromatic compounds in GFBS.

Moreover, acetic acid also had a highly significant effect on the aroma of GFBS. It was previously reported that the strongest odors of bamboo shoots from Taiwan were caused by p-cresol, acetic acid, 2-heptanol, and 1-octen-3-ol, and it is speculated that the main odor may be formed by the synergistic action of p-cresol and acetic acid [5]. Based on the recombination tests and OAV analysis, we speculate that the synergistic effect of p-cresol and acetic acid may be the reason for the main aroma of GFBS. Acetic acid is the most important fatty acid in GFBS and contributes to a strong sour odor. Therefore, acids were also the dominant aromatic compounds in GFBS. Additionally, acetic acid has been reported as the principal aroma-active compound in Japanese miso [29]. The synergistic effect of acetic acid with 3-methylbutanoic acid and 4-methylpentanoic may be the reason for the characteristic sour aroma of PBBP [9].

### 4.2. Subdominant Aroma Compounds in GFBS

As shown in Table 3, five aldehydes are important aroma components of GFBS, specifically, octanal and (E)-2-octenal. (E)-2-octenal exists as a key aroma component, which has also been reported in other foods, such as Millet Huangjiu [31] and Beijing roasted duck [13]. Octanal has been reported to have a significant influence on roasted duck aroma [13]. Most aldehydes are formed via yeast metabolism or carbon chain oxidation of unsaturated fatty acids [32]. Aldehydes in GFBS may be related to yeast fermentation. In terms of alcohols, 13–16 alcohols were detected per GFBS sample, comprising the largest number of compounds in one group. Moreover, in this study, phenylethyl alcohol was identified as the most important alcohol and it exhibited a floral odor, followed by 1-octen-3-ol and 1-nonanol. The important aroma compounds 1-octen-3-ol and phenylethyl alcohol were identified in GFBS, which is consistent with Guo et al. [8]. Alcohols in GFBS are mainly formed through bacterial and fungal fermentation [33].

### 4.3. Other Substances with Important Contributions to GFBS Odor

In this study, ethers, ketones, alkenes, benzene derivatives, and furans were also detected in GFBS. However, they accounted for a small proportion of the total number of compounds, but their contribution is indispensable. Among these, esters are important. A total of 12 esters were detected; ethyl acetate is an important aroma compound, which reportedly plays an important role in the aroma of other fermented foods, such as wine [30]. Esters are mainly formed by the esterase-catalyzed reaction of alcohols and acids produced from glucose and amino acids during microbial metabolism [31].

## 5. Conclusions

This study provided a comprehensive investigation into the identification of potent compounds contributing to the characteristic aroma of GFBS through recombination and omission experiments. A total of 70 aroma compounds were identified by GC-MS, and 18 aroma-active compounds were identified from LZ-GFBS by GC-MS-O. Fifteen aroma-active compounds with OAVs > 1 were further recognized as important aroma compounds. Finally, omission tests further confirmed octanal, (E)-2-octenal, acetic acid, guaiacol, phenylethyl alcohol, creosol, 4-ethylguaiacol, and p-cresol as the key odorants.

## Figures and Tables

**Table 1:** Volatile compounds identified and quantitated in FBS by GC-O-MS.

Compound ^a^	RT ^b^ (min)	RI ^c^		Method ^f^	Concentration ^g,a^			
		Calculated ^d^	Literature ^e^		LZ-FBS	GL-FBS	NN-FBS	BS-FBS
Acetaldehyde	4.097	-	-	MS	66.45 ± 15.01	36.06 ± 5.08	0	0
Propanal	4.765	-	-	MS, S, O	22.06 ± 7.37	9.39 ± 0.9	0	0
Methyl acetate	5.175	818	834	MS	10.58 ± 4.14	0	0	0
Ethyl Acetate	5.97	881	894	MS, RI, S, O	206.49 ± 88.05	57.35 ± 7.27	104.11 ± 28.9	251.16 ± 88
Ethanol	6.822	931	939	MS, RI, S	2107.09 ± 120.17	2052.18 ± 204.02	2719.03 ± 50.77	1930.17 ± 521.31
Propyl acetate	7.611	971	982	MS, RI	213.79 ± 103.65	15.28 ± 8.48	0	0
2-butanol	8.6	1022	1031	MS, RI	159.98 ± 43.24	28.49 ± 2.86	0	0
1-propanol	8.972	1033	1045	MS, RI	1222.33 ± 133	523.13 ± 47.68	0	0
Hexanal	10.211	1085	1081	MS, RI	0	8.4 ± 1.34	133.41 ± 15.37	0
1-butanol	11.607	1142	1147	MS, RI	25.95 ± 5.78	14.15 ± 3.11	0	0
Heptanal	12.824	1191	1194	MS, RI	0	2.96 ± 0.34	14.68 ± 2.29	0
Limonene	13.146	1204	1203	MS, RI	0	0.64 ± 0.29	3.08 ± 0.56	0
3-methyl-1-butanol	13.12	1203	1212	MS, RI	19.68 ± 11.36	23.07 ± 9.02	70.7 ± 3.87	16.55 ± 2.96
4-octanone	13.662	1225	-	MS	5.31 ± 2.36	2.84 ± 1.67	13.19 ± 3.04	3.14 ± 1.71
2-pentyl-furan	13.958	1237	1234	MS, RI	0	0	15.08 ± 3.75	0
1-pentanol	14.165	1245	1254	MS, RI	3.25 ± 0.93	3.31 ± 0.31	14.84 ± 0.82	4.17 ± 0.48
3-methyl-3-buten-1-ol	14.32	1251	1251	MS, RI	1.43 ± 0.27	0	0	2.2 ± 0.4
Styrene	14.596	1263	1264	MS, RI	1.58 ± 0.1	2.6 ± 0.86	2.96 ± 0.82	6.28 ± 1.69
Octanal	15.332	1292	1296	MS, RI, S, O	4.29 ± 1.35	3.54 ± 0.29	12.13 ± 0.43	1.96 ± 1.71
2-heptanol	15.814	1313	1318	MS, RI	22.06 ± 8.91	0	0	0
(E)-2-heptenal	16.224	1330	1332	MS, RI,	3.73 ± 1.85	22.33 ± 10.49	16.9 ± 4.35	11.21 ± 5.05
6-methyl-5-hepten-2-one	16.601	1346	1338	MS, RI	0	2.75 ± 0.07	11.48 ± 2.56	2.35 ± 0.6
Ethyl lactate	16.542	1343	1356	MS, RI	132.13 ± 17.55	60.05 ± 4.31	423.33 ± 10.12	275.68 ± 37.12
1-hexanol	16.639	1348	1361	MS, RI	26.34 ± 8.06	18.64 ± 0.5	82.25 ± 8.59	50.48 ± 6.32
Nonanal	17.937	1403	1400	MS, RI, S, O	8.79 ± 2.2	13.5 ± 2.08	16.44 ± 0.39	5.93 ± 0.76
Ethyl octanoate	18.787	1439	1436	MS, RI	0	1.49 ± 0.38	19.18 ± 15.41	2.24 ± 0.56
1-octen-3-ol	19.015	1448	1452	MS, RI, S, O	8.04 ± 2.42	67.05 ± 8.43	93.01 ± 19.52	13.21 ± 5.36
4-methylanisole	18.956	1446	1441	MS, RI	25.37 ± 15.07	0	0	0
(E)-2-octenal	19.066	1451	1425	MS, RI, S, O	5.81 ± 2.91	2 ± 0.85	17.5 ± 6.56	4.26 ± 2
1-heptanol	19.132	1453	1457	MS, RI	0	19.48 ± 1.91	8.77 ± 1.34	20.34 ± 5.55
Acetic acid	19.572	1472	1473	MS, RI, S, O	3378.09 ± 188.06	2521.16 ± 238.03	3710.7 ± 163.78	2005.91 ± 258.52
2-ethyl-1-hexanol	19.962	1489	1490	MS, RI	0	23.48 ± 1.93	72.38 ± 66.92	150.08 ± 38.07
Benzaldehyde	21.227	1535	1541	MS, RI, S, O	83.51 ± 8.58	94.06 ± 3.13	184.72 ± 123.63	28.87 ± 14.2
Linalool	21.493	1544	1551	MS, RI, S	5.23 ± 3.75	62.14 ± 15.96	28.27 ± 8.29	14.2 ± 12.53
Propanoic acid	21.616	1548	1564	MS, RI, S	402.64 ± 35.87	793.05 ± 32.39	0	0
1-octanol	21.873	1557	1562	MS, RI	14.99 ± 10.99	285.36 ± 387.74	5.65 ± 1.39	2 ± 0.43
Butanoic acid	24.635	1643	1652	MS, RI	18.79 ± 2.34	48.63 ± 2.15	28.39 ± 0.34	0
3-furanmethanol	25.155	1658	-	MS	4.97 ± 0.31	12.57 ± 1.7	4.61 ± 0.07	3.22 ± 0.89
1-nonanol	25.252	1661	1664	MS, RI, S, O	11.1 ± 3.6	10.52 ± 3.2	9.97 ± 4.22	11.85 ± 0.99
Acetophenone	25.447	1666	1671	MS, RI	7.63 ± 1.54	6.79 ± 0.09	21.73 ± 9.79	0
Ethyl benzoate	26.005	1682	1681	MS, RI	9.7 ± 4.27	0	246.73 ± 63.83	0
1,2-dimethoxy-benzene	27.718	1729	1737	MS, RI	138.35 ± 29.34	56.73 ± 1.49	75.86 ± 5.9	32.42 ± 2.68
P-tolyl acetate	28.043	1737	-	MS	20.03 ± 4.81	10.57 ± 4.12	0	18.02 ± 2.42
Naphthalene	28.72	1755	1765	MS, RI, S	5.61 ± 2.68	0	12.95 ± 2.38	0
Methyl salicylate	29.934	1788	1798	MS, RI, S, O	39.04 ± 13.06	15.77 ± 1.94	174.04 ± 40.59	16.84 ± 8.34
3,4-dimethoxytoluene	30.809	1813	1806	MS, RI	25.58 ± 6.45	0	0	0
Ethyl salicylate	31.28	1827	-	MS	0	2.83 ± 0.51	17.75 ± 4.62	0.94 ± 0.85
Hexanoic acid	32.407	1861	1849	MS, RI, S	0	105.1 ± 6.13	504.44 ± 32.38	37.76 ± 2.11
Guaiacol	32.522	1865	1859	MS, RI, S, O	103.44 ± 10.6	120.39 ± 5.45	143.74 ± 1.91	51.22 ± 4.22
Geranylacetone	32.839	1874	1868	MS, RI, S, O	10.21 ± 2.37	5.11 ± 1.9	35.38 ± 30.77	3.94 ± 2.05
Benzyl alcohol	33.012	1880	1880	MS, RI	63.22 ± 9.38	247.65 ± 5.31	187.64 ± 4.95	47.17 ± 4.07
Ethyl hydrocinnamate	33.596	1898	1903	MS, RI	48.79 ± 13.55	25.09 ± 3.83	20.23 ± 3.85	0
Phenylethyl alcohol	34.283	1921	1929	MS, RI, S, O	113.03 ± 22.64	277.87 ± 5.17	153.16 ± 10.71	49.87 ± 5.3
2-chloro-4-methyl-phenol	34.953	1945	-	MS	16.61 ± 11.92	38.17 ± 19.42	0	0
2-ethyl-hexanoic acid	35.48	1964	-	MS	0	21.26 ± 7.59	15.1 ± 3.01	6734.16 ± 446.45
Creosol	35.65	1970	1981	MS, RI, S, O	658.4 ± 104.45	307.7 ± 6.6	363.39 ± 339.52	913.7 ± 58.26
Heptanoic acid	35.641	1969	-	MS	0	0	58.73 ± 5.28	0
Phenol	36.816	2011	2037	MS, RI, S	305.51 ± 31.79	5757.22 ± 54.07	2122.09 ± 20.76	3151.9 ± 115.36
4-ethylguaiacol	37.511	2038	2032	MS, RI, S, O	226.08 ± 49.7	118.08 ± 2.87	69.04 ± 10.95	235.61 ± 20.37
Anisaldehyde	37.905	2053	2058	MS, RI, S, O	6.8 ± 1.43	4.27 ± 1.16	3.49 ± 0.59	2.51 ± 0.59
Octanoic acid	38.543	2077	2084	MS, RI	0	0	300.93 ± 115.1	0
Ethyl tetradecanoate	38.65	2081	-	MS	4.29 ± 3.15	11.33 ± 1.19	0	5.72 ± 4.8
P-cresol	38.814	2087	2085	MS, RI, S, O	16083.21 ± 470.61	19120.68 ± 88.86	15435.89 ± 123.97	21313.09 ± 490.83
4-ethyl-phenol	41.372	2176	2186	MS, RI, S, O	83.35 ± 16.6	96.39 ± 27.33	64.89 ± 6.28	167.54 ± 13.07
Nonanoic acid	41.583	2183	2192	MS, RI	182.91 ± 65.85	369.44 ± 63.03	530.72 ± 335.93	283.5 ± 17.01
Palmitic acid ethyl ester	43.804	2251	2248	MS, RI	122.12 ± 27.93	145.01 ± 94.92	139.31 ± 17.11	192.8 ± 16.95
Myristicin	44.548	2274	2258	MS, RI	0	0	926.59 ± 96.33	0
N-decanoic acid	44.956	2286	2300	MS, RI	0	41.13 ± 8.35	39.16 ± 19.4	44.66 ± 7.63
2,4-di-tert-butylphenol	45.74	2309	2315	MS, RI	284.77 ± 153.05	461.93 ± 48.1	510.82 ± 211.08	406.54 ± 175.48
Benzoic acid	50.683	-	-	MS	0	0	1287.17 ± 292.22	0

^a^ Aroma compounds detected in GFBS. ^b^ Retention time in the capillary GC column. ^c^ Linear retention index. ^d^ Calculated data based on alkanes (C7−C40). ^e^ Reported linear retention index. ^f^ Method of identification: MS, mass spectrum comparison using NIST14; L, library; RI, RI calculation in agreement with literature value; S, confirmed by authentic standards; O, olfactometric confirmation. ^g^ Values represent the peak area ratio of each compound with that of the internal standard. Data are expressed as mean ± standard deviation (*n* = 3).

**Table 2:** Authentic standards, scanned ions, calibration equations, and odor descriptions in the determination of aroma compounds.

Compound	Ions (*m*/*z*) ^a^	Calibration Equation ^b^	R^2^	Odor Description ^c^
Propanal	58.1, 57.1, 44.0, 42.0	y=(0.00002)x+0.1933	0.9905	Pungent
Ethyl acetate	43.1, 61.1, 70.1, 45.1	y=(0.000005)x−1.1427	0.9945	Fruit, wine
Octanal	41.1, 43.1, 57.1, 84.1	y=(0.0000007)+0.0121	0.9983	Citrus, fresh
Nonanal	57.1, 41.1, 55.1, 98.1	y=(0.0000002)x−0.0088	0.9994	Citrus, fatty
1-octen-3-ol	57.1, 43.1, 72.1, 85.1	y=(0.00000008)x+0.0065	0.9989	Green, mushroom
(E)-2-octenal	70.1, 55.1, 41.1, 83.1	y=(0.0000002)x+0.0393	0.9908	Unpleasant, nut, fatty
Acetic acid	43.1, 45.0, 60.1, 42.1	y=(0.0003)x+163.61	0.9980	Vinegar, sour
Benzaldehyde	105.1, 106.1, 77.1, 55.1	y=(0.00000008)x−0.0241	0.9948	Almond, cherry-like
1-nonanol	56.1, 70.1, 41.1, 83.1	y=(0.00000006)x+0.0034	0.9996	Rose, floral
Methyl salicylate	120.1, 92.1, 152.1, 65.1	y=(0.00000001)x+0.0022	0.9906	
Guaiacol	109.1, 124.1, 81.1, 53.1	y=(0.0000002)x−0.01638	0.9997	Smoky, sweet
Geranylacetone	69.1, 43.1, 151.1, 136.1	y=(0.00000003)x+0.00000005	0.9993	Sweet, rose
Phenylethyl alcohol	91.1, 92.1, 122.1, 65.1	y=(0.0000003)x−0.0483	0.9948	Floral, rose, honey
Creosol	138.1, 123.1, 95.1, 67.1	y=(0.0000001)x+0.0049	0.9984	Dusty, woody
4-ethylguaiacol	137.1, 152.1, 122.1, 91.1	y=(0.00000005)x−0.0174	0.9989	Smoky
Anisaldehyde	135.1, 136.1, 77.1, 92.1	y=(0.0000002)x+0.035	0.9960	Phenolic
P-cresol	107.1, 108.1, 77.1, 79.1	y=(0.000001)x−39.412	0.9997	Phenolic, animal
4-ethyl-phenol	107.1, 122.1, 77.1, 91.1	y=(0.00000007)x+0.008	0.9986	Herbal, phenolic

^a^ Monitored ions used for quantification. ^b^ Variables: x is the peak area relative to that of the internal standard, 2-methyl-3-heptanone, and *y* is the concentration (ng/g) in the sample relative to that of the internal standard, 2-methyl-3-heptanone. ^c^ Sniffed odors determined by GC-O analysis.

**Table 3:** Aroma compound OAVs calibrated by standard curve.

Compound	Threshold ^a^ (μg/kg in Water)	LZ	GL	NN	BS
Propanal	60 ^b^	32.83	15.83	0	0
Ethyl acetate	97.8 ^c^	30.83	0.12	9.75	40.03
Octanal	0.7 ^d^	34.56	31.56	66.14	25.17
Nonanal	1.1 ^e^	4.71	8.15	10.31	2.61
1-octen-3-ol	1 ^d^	9.09	28.1	36.46	10.75
(E)-2-octenal	3 ^f^	5.4	3.87	10.12	3.87
Acetic acid	1200 ^b^	3537.23	2674.52	3872.09	2155.79
Benzaldehyde	50 ^b^	0.06	0.12	0.71	0
1-nonanol	2 ^g^	3.04	2.97	2.91	3.13
Methyl salicylate	60 ^h^	0.06	0.05	0.15	0.05
Guaiacol	0.84 ^i^	79.68	95.93	118.32	29.62
Geranylacetone	10 ^b^	0.12	0	0.43	0
Phenylethyl alcohol	53.95 ^e^	1.64	5.33	2.53	0.22
Creosol	8.92 ^e^	30.27	14.44	16.96	41.8
4-ethylguaiacol	1.3 ^j^	21.63	4.91	1.15	23.11
Anisaldehyde	10 ^b^	4.05	3.70	3.78	3.84
P-cresol	3.9 ^e^	38684.24	57826.67	34997.95	71409.51
4-ethyl-phenol	13 ^i^	2.42	2.71	2.02	4.25

^a^ Odor thresholds in water taken from the literature. ^b^ Odor threshold from Van Gemert [22]. ^c^ Odor threshold from Lu et al. [23]. ^d^ Odor threshold from Liu et al. [13]. ^e^ Odor threshold from Zhao et al. [9]. ^f^ Odor threshold from Ren et al. [16]. ^g^ Odor threshold from Rothe et al. [24]. ^h^ Odor threshold from Stevens [25]. ^i^ Odor threshold from Abe et al. [26]. ^j^ Odor threshold from Wang et al. [27].

**Table 4:** Omission tests from complete recombination model.

Odorants Omitted from the Recombination Model	N ^a^	Significance ^b^
Propanal	5	-
Ethyl acetate	4	-
Octanal	6	*
Nonanal	3	-
1-octen-3-ol	4	-
(E)-2-octenal	6	*
Acetic acid	8	***
1-nonanol	3	-
Guaiacol	8	***
Phenylethyl alcohol	7	**
Creosol	7	**
4-ethylguaiacol	6	*
Anisaldehyde	3	-
P-cresol	9	***
4-ethyl-phenol	2	-

^a^ Number of correct judgments from nine panelists; ^b^ * significant (*p* ≤ 0.05); ** highly significant (*p* ≤ 0.01); *** very highly significant (*p* ≤ 0.001).

## Data Availability

The data presented in this study are available on request from the corresponding author.

## Appendix A

**Table A1:** Main characteristics of analyzed Guangxi fermented bamboo shoots samples.

Sample	Species of RAW BAMBOO SHOOTS	Guangxi Fermented Bamboo Shoots Location	Fermentation Time (Month)	pH
LZ-FBS	*Dendrocalamus latiflorus Munro*	Liuzhou	1	3.42 ± 0.02
NN-FBS	*Dendrocalamus latiflorus Munro*	Nanning	1	3.85 ± 0.03
GL-FBS	*Dendrocalamus latiflorus Munro*	Guiling	1	3.93 ± 0.03
BS-FBS	*Dendrocalamus latiflorus Munro*	Baise	1	3.82 ± 0.04
